# A Novel Antigen-Sampling Cell in the Teleost Gill Epithelium With the Potential for Direct Antigen Presentation in Mucosal Tissue

**DOI:** 10.3389/fimmu.2018.02116

**Published:** 2018-09-20

**Authors:** Goshi Kato, Haruya Miyazawa, Yumiko Nakayama, Yuki Ikari, Hidehiro Kondo, Takuya Yamaguchi, Motohiko Sano, Uwe Fischer

**Affiliations:** ^1^Department of Marine Biosciences, Tokyo University of Marine Science and Technology, Tokyo, Japan; ^2^Institute of Infectology, Friedrich-Loeffler-Institute, Federal Research Institute for Animal Health, Greifswald-Insel Riems, Germany

**Keywords:** *Oncorhynchus mykiss*, comparative immunology, mucosal vaccination, atypical antigen presenting cell, lower vertebrate

## Abstract

In mammals, M cells can take up antigens through mucosal surfaces of the gut and the respiratory tract. Since M cells are deficient of lysosomes and phagosomes, the antigens are directly delivered to the mucosa-associated lymphoid tissue (MALT) without degradation. In teleost fish, the entire body surface (gills, skin, and intestinal system) is covered by mucus; however, specific antigen-sampling cells have not yet been identified in their mucosal tissues. Here, we show that two phenotypes of antigen-sampling cells take up antigens through epithelial surfaces of the rainbow trout gill. One phenotype of antigen-sampling cells has features of monocyte/macrophage/dendritic cell-type cells; they have large vacuoles in the cytoplasm and express PTPRC (CD45), CD83, IL-1β, and IL-12p40b. The second phenotype exhibits similar characteristics to mammalian M cells; the corresponding cells bind the lectin UEA-1 but not WGA and show expression of M cell marker gene Anxa5. In contrast to mammalian M cells, teleost M-type cells were found to exhibit small vacuoles in their cytoplasm and to express almost all genes related to the “phagosome”, “lysosome,” and “antigen processing and presentation” pathways. Furthermore, MHC class II was constitutively expressed on a fraction of M-type cells, and this expression was significantly increased after antigen uptake, suggesting that the MHC class II is inducible by antigen stimulation. Here, we suggest that teleost M-type cells play a role in the phylogenetically primitive teleost immune system, similar to bona-fide M cells. In addition, the presence of MHC class II expression suggests an additional role in antigen presentation in the gills, which are an organ with high T cell abundance, especially in interbranchial lymphoid tissue. The present results suggest an unconventional antigen presentation mechanism in the primitive mucosal immune system of teleosts, which generally lack highly organized lymphoid tissues. Moreover, the results of this work may be valuable for the development of mucosal vaccines that specifically target M-type cells; mucosal vaccines significantly reduce working costs and the stress that is usually induced by vaccination via injection of individual fish.

## Introduction

The mucosal surfaces of the digestive and respiratory systems are continuously exposed to the external environment and therefore represent potential ports of pathogen entry. The immune system has evolved to prevent pathogen entry in mucosal tissues, and if an entry is unavoidable, to mount a local immune response. In mammals, recognition of pathogens, and initialization of the immune response occurs in mucosa-associated lymphoid tissue (MALT). MALT consists of germinal centers, lymphoid follicles, and T cell regions and harbors professional antigen-presenting cells (APCs), such as macrophages and dendritic cells (1). M (membranous epithelial, microfold, or microvillous) cells (2) are atypical epithelial cells that phagocytize antigens and macromolecules in the follicle-associated (dome) epithelium (FAE) of gut-associated lymphoid tissue (GALT) and nasopharynx-associated lymphoid tissue (NALT) in mammals. In mice, the lectin *Ulex europaeus* agglutinin-1 (UEA-1), which specifically binds to α (1, 2) fucose and it has been established as an excellent marker for human endothelial cells, is routinely used to identify M cells. In contrast, M cells do not test positive for the lectin wheat germ agglutinin (WGA), which binds to UEA-1^+^ goblet cells in FAE (3). Molecules on the surface of M cells such as glycoprotein 2 (4), integrin β1 (5), and α2-3-linked sialic acid (6) have been identified as receptors involved in the uptake of FimH^+^ bacteria, *Yersinia enterocolitica* and type 1 reovirus, respectively. Following their capture by the corresponding receptors, M cells mainly transcytose the respective antigens and deliver them to subjacent APCs (7), and the APCs then present antigens to T lymphocytes in MALT. Finally, antigen-specific immune responses, such as production of IgA by B cells, are induced in mucosal tissues.

Fish inhabit aquatic environments, in which microorganisms are more abundant than in terrestrial environments. The entire body surface of fish (gills, intestine, and skin) is covered by mucus, which is one of the initial immune barriers preventing the invasion of pathogens. Unlike mammals, teleost fish lack lymphoid structures such as germinal centers, B-cell follicles, lymph nodes, and structured MALT. Zapata and Amemiya (8) described the teleost GALT as diffuse subepithelial lymphoid aggregates. Another lymphoid structure that complies with the definition of a tissue is found in the gill epithelium and is referred to as interbranchial lymphoid tissue (ILT). Although the function of ILT is yet to be elucidated, it is considered to represent a phylogenetically early form of leukocyte accumulation in a respiratory organ (9–11). Another special feature of teleost fish is the production of a unique immunoglobulin, IgT, that is suggested to be specialized for mucosal immunity and to possess similar functions to mammalian IgA, although IgT, and IgA are phylogenetically distant immunoglobulins (12).

Mucosal delivery of vaccines, for example, via immersion or oral immunization, is the preferred vaccination method for preventing infectious diseases in aquaculture (13). These vaccination methods significantly decrease the working cost of vaccination in aquaculture since they are suitable approaches for mass vaccination. Vaccine antigens that are administered via the oral route are taken up by the intestinal epithelium of teleost fish (14). The first evidence for the existence of M cells in fish was found in rainbow trout, in which the M-like cells were shown to exhibit similar characteristics to mammalian M cells, exemplified by their morphology (with openly arranged microvilli) and their affinity for the lectin UEA-1 but not WGA (15). In zebrafish, M-like cells have not been yet described, but nanoparticles, and bacteria (*Mycobacterium marinum*) are taken up in the intestinal epithelium when administered via an oral route (16). Bath vaccination of teleost fish is effective against pathogenic bacteria such as *Aeromonas salmonicida* subsp. *salmonicida* (*Ass*) (17), *Vibrio anguillarum* (18), and *Yersinia ruckeri* (19). Large numbers of fish are dipped into a vaccine solution that is traditionally composed of formalin-killed bacteria. While soluble antigens in the vaccine solution are mainly taken up via the skin (20), particulate antigens, such as bacterins from *Y. ruckeri*, are taken up not only via the gill epithelium but also via the gastrointestinal tract, probably after ingestion of the vaccine solution (21, 22). Kato et al. (23) showed that *V. anguillarum* bacterin was taken up primarily via gill epithelial cells, inducing the up-regulation of inflammatory cytokine genes. However, little is known about the exact antigen-sampling mechanisms in the gill epithelium of teleost fish or about the cell populations involved and the resulting local and systemic immune responses.

In this study, we identified and characterized two types of antigen-sampling cells in the rainbow trout gill epithelium that are involved in bacterin uptake during bath vaccination: resident DC/macrophage-type cells in the gill epithelium and another group of antigen-sampling cells that exhibit phenotypic characteristics of M cells, expressing MHC class II molecules on their surface. The M-type antigen-sampling cells showing MHC class II expression were significantly increased in the gill epithelium after bath vaccination. Thus, we hypothesize that antigen-sampling cells are involved in direct antigen presentation to T cells in the gill mucosal tissue of teleosts, which lack highly organized lymphoid organs.

## Materials and methods

### Fish rearing and bacterial propagation

Rainbow trout (56–126 g body weight) were reared in 400 L tanks at 15°C in a recirculating water system at the Friedrich-Loeffler-Institut and the Tokyo University of Marine Science and Technology. Fish were anesthetized with benzocaine or 2-phenoxyethanol prior to dissection. All animal experiments were approved by the Institutional Animal Care and Use Committee in TUMSAT and FLI. *Ass* bacteria isolated from diseased rainbow trout were cultured in veal infusion broth (VIB) or tryptic soy broth (TSB). *V. anguillarum* (serotype J-O-1, J-O-2, and J-O-3) isolated from diseased ayu *Plecoglossus altivelis* were cultured in TSB. The cultured bacteria were inactivated in 0.3% formalin overnight and then washed.

### Immunohistochemistry

Rainbow trout were dipped into a vaccine solution initially corresponding to 2.6–4.4 × 10^8^ CFU/ml of *Ass* bacterin, followed by incubation for 30 min. The gills of the bath-vaccinated fish were fixed overnight in Davidson's solution, embedded in paraffin and sectioned at a 3 μm thickness. The sections were subsequently incubated with polyclonal rabbit antiserum raised against *Ass* (1:1000 dilution) for 1 h at 4°C. After being washed three times with 1 × tris buffered saline (TBS), the sections were treated with a VECTASTAIN ABC Rabbit IgG kit (VECTOR Laboratories) following the manufacturer's instructions. Next, the sections were washed again, treated with 50 mM Tris-HCl (pH 7.6) containing 0.02% 3,3′-diaminobenzidine tetrahydrochloride (Dojindo) and 0.03% H_2_O_2_ for 3 min, and counterstained with hematoxylin. For immunofluorescence assays, sections stained with the anti-*Ass* serum were incubated with a UEA-1 TRITC conjugate (final concentration, 10 μg/ml; Sigma-Aldrich) or WGA rhodamine conjugate (10 μg/ml, VECTOR Laboratories) for 30 min at 4°C. A goat anti-rabbit IgG (H+L) Alexa Fluor 488 conjugate (4 μg/ml, Thermo Fisher Scientific) was used as a secondary antibody to detect the *Ass* bacterin. Paraffin sections of gills from unvaccinated fish were also stained with UEA-1 FITC conjugate (10 μg/ml, Sigma-Aldrich) and WGA rhodamine conjugate, as above. Cryosections of gills sampled 3 h after *ex vivo* bath vaccination were fixed with acetone for 10 min and incubated with an anti-rainbow trout MHC class II mAb (24) (a gift from Dr. C. Tafalla) for 30 min at 4°C. The slides were then washed three times and incubated with goat anti-mouse IgG (H+L) Alexa Fluor 555 (2 μg/ml, Thermo Fisher Scientific) for 30 min at 4°C. The sections were finally stained with Hoechst 33342 (Thermo Fisher Scientific) and mounted with ProLong Gold Antifade Mountant (Thermo Fisher Scientific), and digital images were captured and analyzed with a Nikon ECLIPSE Ti S fluorescence microscope and NIS Elements software (Nikon).

### Flow cytometry

Formalin-inactivated *Ass* and *V. anguillarum* J-O-1, J-O-2, and J-O-3 were stained with SYTO 61 (Thermo Fisher Scientific). The gills were treated with bacterins *ex vivo* as described by Torroba et al. (25). Briefly, the gills were removed from anesthetized naïve fish and washed twice using RPMI 1640 medium for 5 min with a stirring at 400 rpm. Four pieces of the gills were dipped into SYTO 61-stained bacterin (1.0–2.0 × 10^8^ CFU/ml) or 1.0 × 10^7^ particles/ml of Fluoresbrite YG Carboxylate microspheres (0.97 μm, Polysciences) suspended in RPMI 1640 medium (Nissui) for 30 min with a stirring at 400 rpm. The other four pieces of the gills were incubated in RPMI 1640 as above and used as a negative control. It was confirmed that the data obtained using the *ex vivo* method were equivalent to those from *in vivo* bath vaccination.

After *ex vivo* bath vaccination with SYTO 61-stained bacterin, gills were washed twice with RPMI 1640 medium. For separation of the gill epithelial cells, the gills were incubated in PBS containing 10 mM EDTA with a stirring at 400 rpm for 20 min at 4°C. The cells were then washed three times with RPMI 1640, counted, and resuspended in RPMI 1640 at 0.84–1.64 × 10^8^ cells/ml (>85% viability). One-hundred microliter of the cell suspension was incubated with FITC-labeled UEA-1 diluted in the cell culture medium (10 μg/ml). Further, UEA-1-treated cells were also stained with a mAb against rainbow trout MHC class II (1: 1,000 dilutions in the cell culture medium) for 1 h at 4°C, and a goat anti-mouse IgG PE/Cy 5.5 conjugate (0.25 μg/ml, Abcam) diluted in RPMI 1640 was used as secondary antibodies. The epithelial cells from the gills dipped into the microspheres were stained with UEA-1 (10 μg/ml in RPMI 1640, without fluorescent conjugation). A rabbit anti-UEA-1 polyclonal antibody (5 μg/ml in RPMI 1640, Bioss Antibodies) and goat anti-rabbit IgG (H+L) Alexa 647 (2 μg/ml in RPMI 1640, Abcam) were used as secondary and tertiary antibodies to detect UEA-1, respectively. Afterbeing washed three times with RPMI 1640, the cells were re-suspended in the cell culture medium and stained with DAPI (4',6-diamidino-2-phenylindole, 1 μg/ml in RPMI 1640; Life Technologies). The epithelial cells collected from the gills exposed to RPMI 1640 were incubated with the secondary antibodies and used as negative controls. Flow cytometry analyses of 10,000 cells in each group were performed using a CytoFlex (Beckman Coulter). Data from the cytometers were analyzed with Kaluza Flow Cytometry Analysis Software (Beckman Coulter).

### Cell sorting by flow cytometry

Formalin-inactivated *Ass* were stained with SYTO 61 (Thermo Fisher Scientific) and used for bath vaccination. Rainbow trout were dipped into a vaccine solution initially corresponding to 1.0–2.0 × 10^8^ CFU/ml of SYTO 61-stained bacterins, followed by incubation for 30 min. After *in vivo* bath vaccination with SYTO 61-stained bacterin, gills were washed twice with the ZB28 cell culture medium (50/50 mixture of Iscove's and Ham's F12 medium). The gill epithelial cells were prepared and stained with FITC-labeled UEA-1 (10 μg/ml in ZB28), as above. After being washed three times with ZB28, the cells were re-suspended in the cell culture medium and stained with propidium iodide (PI, 2 μg/ml; Life Technologies) or DAPI (4',6-diamidino-2-phenylindole, 1 μg/ml; Life Technologies). The gill epithelial cells collected from rainbow trout dipped into rearing water without SYTO61-stained bacterin were prepared and used as negative control. Cell sorting was performed with a MoFlo high-speed cell sorter (Dako Cytomation) or a FACSAria Fusion cell sorter (BD Biosciences). First, PI^+^ or DAPI^+^ cells were gated to exclude sorting dead cells. Then, 30,000 cells of UEA-1^−^ *Ass*^−^ population as a negative control, UEA-1^−^ *Ass*^+^ population and UEA-1^+^ *Ass*^+^ population were sorted. Data from the cytometers were analyzed with Kaluza Flow Cytometry Analysis Software (Beckman Coulter).

### Gene expression analysis

Total RNA was extracted from sorted cells using a NucleoSpin RNA XS kit (Machery-Nagel), following the manufacturer's instructions. Equal amounts of total RNA from each sorted cell population of three fish individuals were pooled prior to library construction. RNA libraries were constructed using the Illumina TruSeq Stranded mRNA Sample Preparation Kit (Illumina), following the manufacturer's instructions. Sequencing was performed using the Illumina MiSeq platform, employing the MiSeq Reagent Kit v2 (Illumina), with 151 paired-end reads. The whole transcriptome sequence raw data were deposited in the DDBJ Sequence Read Archive (Accession Number: DRA006692). *De novo* assembly of the MiSeq reads and gene expression analysis using the fragments per kilobase of exon per million mapped reads (FPKM) model was performed with Trinity v2.1.1 software (https://github.com/trinityrnaseq/trinityrnaseq). The assembled sequences were annotated using the BLAST program at the UniProtKB database (http://www.uniprot.org/help/uniprotkb) and using Blast2GO software (https://www.blast2go.com/). The UniProtKB accession numbers of the expressed genes of each library were submitted to Venny 2.1 software (http://bioinfogp.cnb.csic.es/tools/venny/) to construct a Venn diagram. The KAAS (http://www.genome.jp/tools/kaas/) was employed for orthologue assignment and pathway mapping.

Total RNA was extracted from each sorted cell population using Nucleospin RNA XS kit (Machery-Nagel), following manufacturer's instructions. First-strand cDNAs were synthesized using 100 ng of total RNA using M-MLV reverse transcriptase (Thermo Fisher Scientific). The primers employed for qRT-PCR are shown in Table S3. The expression levels of several genes of interest in each sorted cell population from three individuals were analyzed by quantitative RT-PCR (qRT-PCR) using THUNDERBIRD SYBR qPCR Mix (TOYOBO), following the manufacturer's instructions. Since the PCR efficiency of all primer sets for qPCR were higher than 95%, the relative values for expression levels were calculated using the 2^−ΔΔCt^ method, taking into account the expression of elongation factor 1α (Accession No. AF498320) as an internal control. Significant differences among samples were assessed using one-way analysis of variance (ANOVA) and Tukey's *post-hoc* test. *P* < 0.05 were considered statistically significant.

## Results

### Identification of two phenotypes of antigen-sampling cells in the gill epithelium of rainbow trout

**Figure 1:**
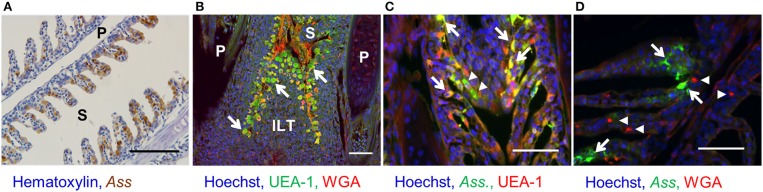
Bacterin uptake by UEA-1^+^ and UEA-1^−^ gill epithelial cells after bath vaccination. **(A)** Immunohistochemistry for the detection of *Ass* bacterin in paraffin section of gills from bath-vaccinated fish. Scale bars, 100 μm. **(B)** Lectin staining in a paraffin gill section. White arrows indicate UEA-1^+^ WGA^−^ cells. Scale bars, 50 μm. **(C)** Fluorescent staining with an anti-*Ass* polyclonal antibody (in green) and the lectin UEA-1 (in red) in gill paraffin section from bath-vaccinated fish. White arrows and white arrowheads indicate UEA-1^+^ and UEA-1^−^ antigen-sampling cells, respectively. Scale bars, 50 μm. **(D)** Fluorescent staining with an anti-*Ass* polyclonal antibody (in green) and the lectin WGA (in red) in gill paraffin section from bath-vaccinated fish. White arrows and arrowheads indicate antigen-sampling cells and WGA-stained cells, respectively. Scale bars, 50 μm. P, primary lamellae; S, secondary lamellae; ILT, interbrachial lymphoid tissue. Experiments were done by *in vivo* bath vaccination and data are from one experiment **(A)** and representative of three experiments **(B–D)**.

**Figure 2:**
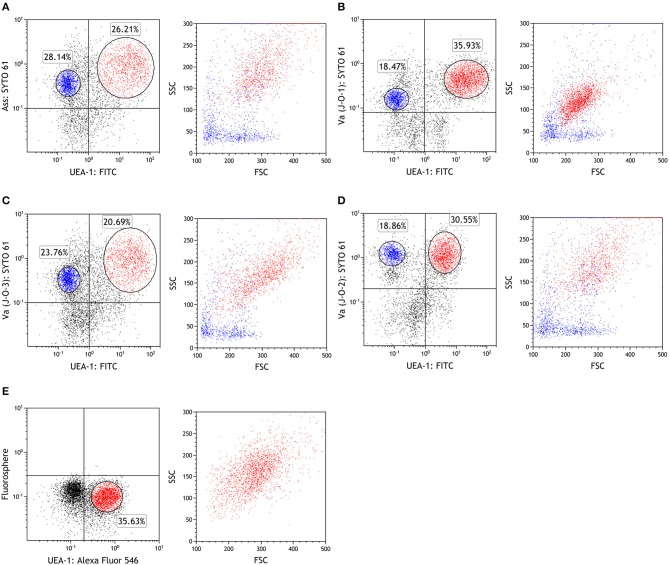
UEA-1^+^ and UEA-1^−^ gill epithelial cells take up bacterins but not microbeads during bath administration. **(A–D)** Flow cytometry of fish gill epithelial cells bath vaccinated with *Ass* bacterin in **(A)**, with *V. anguillarum* J-O-1 serotype bacterin in **(B)**, with *V. anguillarum* J-O-2 serotype bacterin in **(C)**, and with *V. anguillarum* J-O-3 serotype bacterin in **(D)**. **(E)** Flow cytometry of gill epithelial cells from fish immersed with fluorescent microbeads (diameter: 1 μm). Left panels show dot plots of depicting antigen or bead staining on the Y-axis against UEA-1 staining on the X-axis. Red dots represent UEA-1^+^ bacterin^+^ cells, while blue dots represent UEA-1^−^ bacterin^+^ cells in the right panels. Experiments were done by *ex vivo* bath vaccination and data are representative of two experiments.

Since in antigen sampling in rainbow trout has been studied only using live *Ass* (26), and the site of *Ass* bacterin uptake is still unknown, we first aimed to determine the localization of the respective antigen-sampling cells. Immunohistochemistry analysis showed that inactivated *Ass* bacteria were frequently taken up by gill epithelial cells after bath vaccination (Figure 1A). Lectin staining revealed numerous UEA-1^+^ WGA^−^ cells in the epithelial cells of the primary and secondary lamellae. This UEA1^+^ WGA^−^ phenotype, which is typical of mouse M cells, was found both in the epithelia of the outer lamellae and close to the base of the gill filament's septae, where ILT is located (Figure 1B). UEA-1^+^ WGA^+^ staining, which is typical of mammalian goblet cells, was scarcely observed in the gill epithelial cells of rainbow trout. Double staining with an anti-*Ass* polyclonal antibody and either of the lectins after bath vaccination revealed that both UEA-1^+^ and UEA-1^−^ cells were able to take up *Ass* bacterin (Figure 1C), while WGA^+^ cells were not (Figure 1D). To further characterize the antigen-sampling cells, gill epithelial cells were isolated and analyzed via flow cytometry. Similar to the immunohistochemistry analysis, flow cytometry revealed two cell populations that take up *Ass* bacterin in the gill epithelium: UEA-1^+^ cells and UEA-1^−^ cells (Figure 2A left panel). These two populations showed distinct side scatter characteristics, with higher side scatter being observed in UEA-1^+^ *Ass*^+^ cells than in UEA-1^−^ *Ass*^+^ cells, suggesting that UEA-1^+^ *Ass*^+^ cells are more highly granulated than UEA-1^−^ *Ass*^+^ cells (Figure 2A right panel). Since Kato et al. (23) showed previously that *V. anguillarum* bacterin is taken up by the gill epithelium in bath-vaccinated Japanese flounder, we further analyzed gill epithelial cells of rainbow trout that were bath vaccinated with *V. anguillarum* (J-O-1, J-O-2, and J-O-3 serotype) bacterin. Similar to *Ass* bacterin, *V. anguillarum* bacterin was taken up by UEA-1^+^ cells (Figures 2B–D left panels). Additionally, UEA-1^−^ *V. anguillarum*^+^ cells were observed in the gill epithelium of fish that were bath vaccinated with *V. anguillarum* bacterin (Figures 2B–D right panels). However, fluorescence microbeads were taken up neither by UEA-1^+^ nor UEA-1^−^ cells (Figure 2E). In addition, the gill epithelial cells that took up *Ass* bacterin (Figure S1A) did not react with mAbs against CD8α, IgM, and thrombocytes (Figures S1B–D). Based on these results, we identified two cell populations that take up bacterins in the gill epithelium of bath-vaccinated rainbow trout.

### Characterization of the two phenotypes of antigen-sampling cells

**Figure 3:**
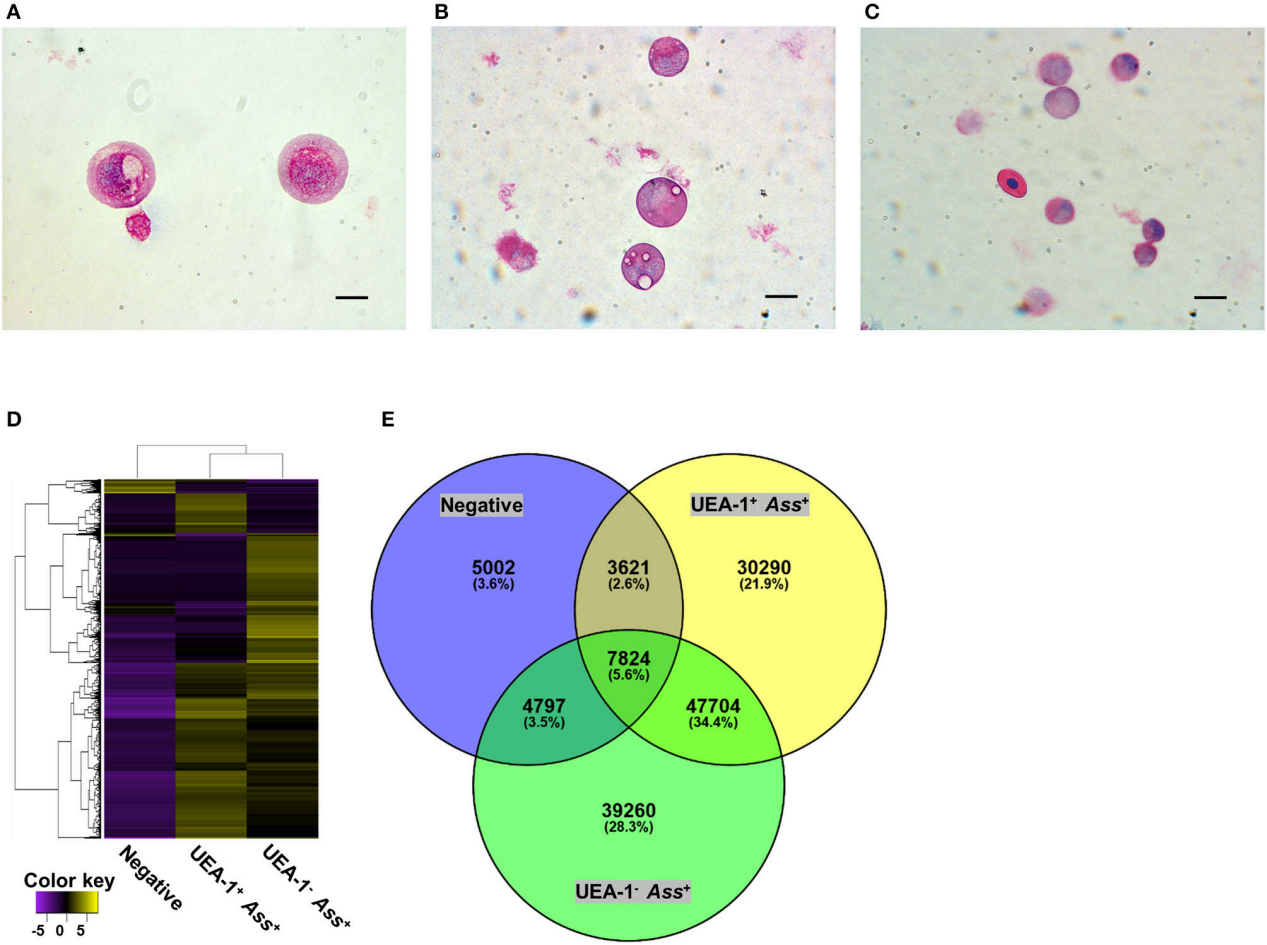
The two antigen-sampling cell populations in the gill epithelium show distinct morphologies and gene expression profiles. **(A–C)** May-Grünwald-Giemsa staining of sorted UEA-1^+^ *Ass*^+^ cells in **(A)**, UEA-1^−^ *Ass*^+^ cells in **B**, and negative cell population in **(C)**. scale bars, 10 μm. **(D)** Heat map of differentially expressed genes in each sample, constructed with the Trinity program. **(E)** Venn diagram summary of differentially expressed genes in the UEA-1^+^ *Ass*^+^, UEA-1^−^ *Ass*^+^ and negative cell populations. Experiments were done by *in vivo* bath vaccination and data are representative of two experiments **(A–C)** or come from one experiment involving three individuals **(D,E)**.

**Figure 4:**
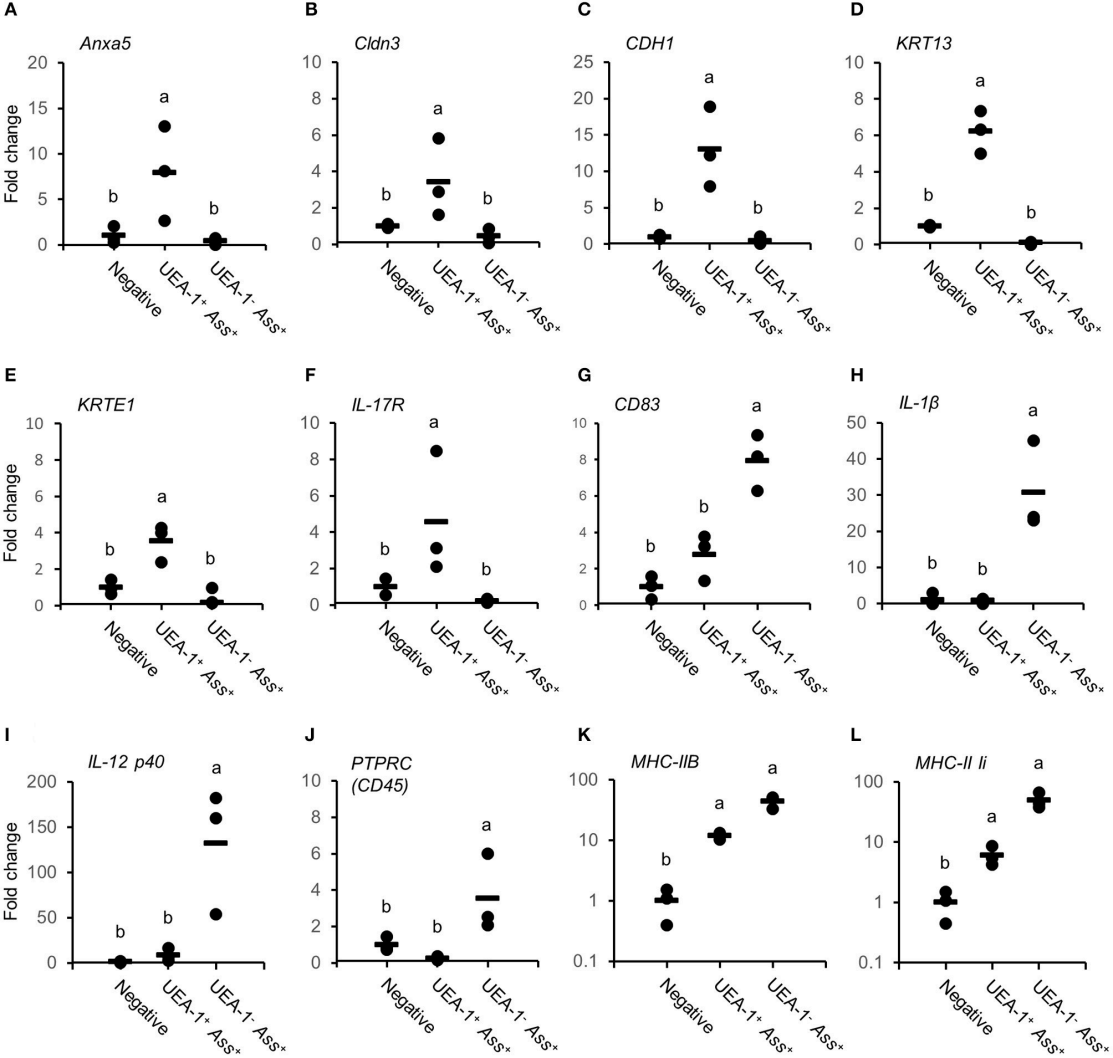
Expression levels of genes expressed in UEA-1^+^ *Ass*^+^ and in UEA-1^−^ *Ass*^+^ cells. **(A–L)** Gene expression levels of *Anxa5* in **(A)**, *Cldn3* in **(B)**, *CDH1* in **(C)**, *KRT13* in **(D)**, *KRTE1* in **(E)**, *IL-17R* in **(F)**, *CD83* in **(G)**, *IL-1*β*3* in **(H)**, *IL-12p40b* in **(I)**, *PTPRC (CD45)* in **(J)**, *MHC-IIB* in **(K)**, and *MHC-II Ii* in **(L)**, in the three sorted cell populations after bath vaccination. Each dot indicates the gene expression level relative to the mean expression level in the negative cell population, which was set as 1. Horizontal lines indicate the mean expression levels (*n* = 3). Different letters represent significant differences between cell populations (*p* < 0.05, one-way ANOVA with Tukey's *post-hoc* test). Experiments were done by *in vivo* bath vaccination and Data are from one experiment with three individual fish.

To characterize the two antigen-sampling cell populations in the gill epithelium, the UEA-1^+^ *Ass*^+^ and UEA-1^−^ *Ass*^+^ cell populations and the negative cell population were sorted via flow cytometry (Figure S2). May-Grünwald-Giemsa (MGG) staining of the sorted cells confirmed that UEA-1^+^ *Ass*^+^ cells exhibited round nuclei, with reddish-purple granules and small vacuoles in the cytoplasm (Figure 3A). Most of the UEA-1^−^ *Ass*^+^ cells showed typical monocyte/macrophage morphologies, including oval nuclei with a homogenous chromatin pattern and pale-blue cytoplasm with vacuoles (Figure 3B). Negative cell population included many cell phenotypes such as erythrocytes, monocyte-like cells and others (Figure 3C). Total RNA samples from the sorted cells were subjected to transcriptome analysis. Deep sequencing yielded 174,245 contigs from sixty-five million total reads (Table S1) and revealed clear differences in gene expression patterns among the three cell populations (Figure 3D). Venn diagrams of the three RNA libraries showed that there were 5,002, 30,290, and 39,260 unique genes expressed in the negative, UEA-1^+^ *Ass*^+^ and UEA-1^−^ *Ass*^+^ cell populations, respectively, (Figure 3E). Pathway analysis using the Kyoto Encyclopedia of Genes and Genome Automatic Annotation Server (KAAS) revealed that the RNA libraries from both UEA-1^+^ *Ass*^+^ and UEA-1^−^ *Ass*^+^ cells contained an almost full set of genes related to the “lysosome,” “phagosome,” and “antigen processing and presentation” pathways (Table S2, and Figures S3, S4). Genes of interest that were highly expressed in UEA-1^+^ *Ass*^+^ and UEA-1^−^ *Ass*^+^ cells are shown in Table 1. Several genes encoding epithelial cell markers, *CDH1, Cldn3, KRT13, KRTS8, KRTE1, KRTE3*, and *IL-17R*, were highly expressed in UEA-1^+^ *Ass*^+^ cells. Several genes that are highly expressed in mammalian M cells, such as *Anxa5, Ctsh, CCL20, Dnclc*, and *Gpx I*, were highly expressed in UEA-1^+^ *Ass*^+^ cells as well. However, UEA-1^+^ *Ass*^+^ cells also expressed *MHC-IIA, MHC-IIB*, and *MHC-II Ii* at high levels. Macrophage, monocyte and dendritic cell makers such as *CD83, IL-1*β, *IL-10R*, and *IL-12 p40* were highly expressed in the UEA-1^−^ *Ass*^+^ cell population, while these genes were not expressed or negligibly expressed in UEA-1^+^ *Ass*^+^ cells. Gene expression levels determined via MiSeq sequencing in the negative, UEA-1^+^ *Ass*^+^ and UEA-1^−^ *Ass*^+^ cell populations were confirmed using qRT-PCR. The expression level of the M cell marker gene *Anxa5* was significantly higher in UEA-1^+^ *Ass*^+^ cells than in the negative and UEA-1^−^ *Ass*^+^ cell populations (*p* < 0.05, Figure 4A). The epithelial cell markers *Cldn3, CDH1, KRT13, KRTE1*, and *IL-17R* also showed significantly higher expression in UEA-1^+^ *Ass*^+^ cells than in the other two cell populations (*p* < 0.01, Figures 4B–F). In contrast, the gene expression levels of *CD83, IL-1*β, and *IL-12 p40* were significantly higher in UEA-1^−^ *Ass*^+^ cells (*p* < 0.05, Figures 4G–I). In addition, the gene expression level of *PTPRC* (*CD45*), a lineage maker for hematopoietic origin, was significantly higher in UEA-1^−^ *Ass*^+^ cells (*p* < 0.05, Figure 4J). qRT-PCR analyses further confirmed the significantly higher expression level of *MHC-IIB* and *MHC-II Ii* in UEA-1^+^ *Ass*^+^ cells and UEA-1^−^ *Ass*^+^ cells than in the negative cell population (*p* < 0.05, Figures 4K–L). Despite the high mRNA levels of MHC class II-related genes observed in UEA-1^+^ *Ass*^+^ cells, we conclude that these cells do not belong to the macrophage/monocyte/dendritic cell lineage but to a cell type that is reminiscent of M cells. The second class of antigen-sampling cells, the UEA-1^−^ *Ass*^+^ cell population, is suggested to be gill epithelium-resident macrophages, monocytes or dendritic cells.

**Table 1:** Genes of interest expressed in UEA-1^+^ Ass^+^ cell and UEA-1^−^ Ass^+^ cell population.

**Description (gene symbol)**	**Accession (UniProtKB)**	**Blast Hit Acc (nr), Matched species**	***E*-value**	**FPKM**		
				**negative**	**UEA-1^+^ Ass^+^**	**UEA-1^−^Ass^+^**
**EPITHELIAL CELL**						
Claudin 3 (*Cldn3*)	A0A060WMH8_ONCMY	XP_013985100, *S. salar*	2.2E-30	0.0	119.8	26.7
E-cadherin (*CDH1*)	M1VNS4_ONCMY	BAM84263, *O. mykiss*	6.0E-71	0.0	154.1	34.8
Interleukin-17 receptor precursor (*IL-17R*)	A1IMH6_ONCMY	NP_001117885, *O. mykiss*	7.5E-35	35.5	111.2	0.0
Type I keratin13 (*KRT13*)	K1C13_ONCMY	NP_001117848, *O. mykiss*	2.0E-153	22.7	99.5	17.5
Type I keratin S8 (*KRTS8*)	Q90W73_ONCMY	NP_001117826, *O. mykiss*	0.0E+00	0.0	354.8	106.7
Type II keratin E1 (*KRTE1*)	Q90W76_ONCMY	NP_001118215, *O. mykiss*	0.0E+00	0.0	151.1	33.2
Type II keratin E3 (*KRTE3*)	Q8JFG4_ONCMY	NP_001123458, *O. mykiss*	0.0E+00	0.0	587.7	198.9
**M CELL**						
Annexin A5 (*Anxa5*)	A0A060W4V6_ONCMY	NP_001134508, *S. salar*	0.0E+00	0.0	222.2	66.7
Cathepsin H (*CtsH*)	A0A060YPS5_ONCMY	XP_021427100, *O. mykiss*	0.0E+00	69.4	412.6	999.5
C-C motif chemokine 20 (*CCL20*)	A0A060Z5E6_ONCMY	XP_020314768, *O. mykiss*	3.8E-50	0.0	325.5	88.4
Dynein light chain cytoplasmic (*Dnclc*)	A0A060W6Y3_ONCMY	XP_021477488, *O. mykiss*	5.4E-62	0.0	91.4	46.9
Glutathione peroxidase 1 (*GpxI*)	W8W106_ONCMY	CCG28020, *O. mykiss*	4.7E-135	0.0	177.1	72.4
**MACROPHAGE AND DENDRITIC CELL**						
CD83	Q6WZ85_ONCMY	AAP93909, *O. mykiss*	6.1E-23	0.0	63.12	1181.6
Interleukin-1 beta 3 (*IL-1β3*)	A0A060YIQ8_ONCMY	CAD89533, *O. mykiss*	0.0E+00	0.0	11.6	107.9
Interleukin-10 receptor subunit beta (*IL-10R*)	A0A060ZFQ5_ONCMY	XP_020338312, *O. kisutch*	0.0E+00	16.2	2.02	85.3
Interleukin 12 subunit beta (*IL-12 p40b*)	X5J4B0_ONCMY	CCH10517, *O. mykiss*	0.0E+00	3.3	0.0	390.5
MHC class II alpha partial (*MHC-IIA*)	E0WN08_ONCMY	CBX11163, *O. mykiss*	7.5E-57	0.0	723.1	1995.4
MHC class II beta partial (*MHC-IIB*)	E0WN21_ONCMY	CBX11176, *O. mykiss*	0.0E+00	0.0	1732.4	3760.0
MHC class II invariant chain (*MHC-II Ii*)	Q8MHB9_ONCMY	AAL91669, *O. mykiss*	1.2E-143	0.0	1241.8	8911.1

### MHC class II expression in the two phenotypes of antigen-sampling cells

**Figure 5:**
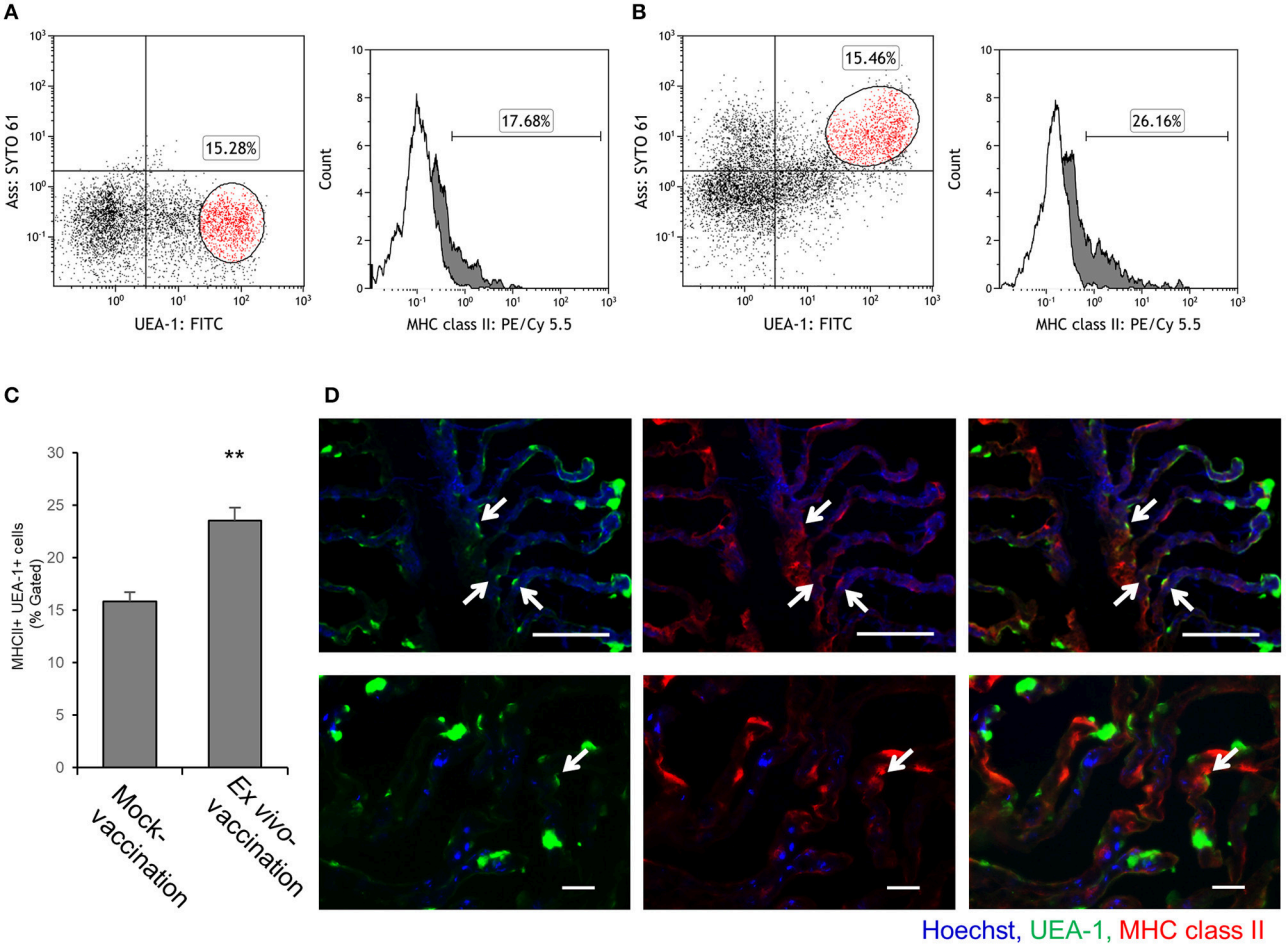
MHC class II expression on UEA-1^+^ cells after *ex vivo* bath vaccination with *Ass* bacterin. **(A,B)** Flow cytometry for UEA-1, *Ass* and MHC class II staining of gill epithelial cells from the gills *ex vivo* bath-vaccinated with *Ass* bacterin in **A** and mock-vaccinated in **B**. Left panels show dot plots for UEA-1 and *Ass* staining of gill epithelial cells. Right panels show the percentage of MHC class II^+^ cells, gated on the UEA-1^+^ cell population. White areas show cells treated only with secondary antibodies (conjugate control), and gray areas show cells positive for MHC class II. **(C)** Mean values for the percentage of MHC class II^+^ cells gated for UEA-1^+^ gill epithelial cells of mock-vaccination and *ex vivo* bath*-*vaccination (*n* = 3). ** *p* < 0.01, Student's *t*-test (two-sided). **(D)** MHC class II^+^ UEA-1^+^ gill epithelium cells in the gills *ex vivo* bath-vaccinated with *Ass* bacterin in lower magnification (upper panels) and higher magnification (lower panels). Arrows indicate MHC class II^+^ UEA-1^+^ cells. P, primary lamellae; S, secondary lamellae; scale bar, 50 μm (upper panels) and 20 μm (lower panels). Experiments were done by *ex vivo* bath vaccination. Data are representative of three experiments **(A,B)**, or one experiment with three individuals **(C)**, or representative of three experiments **(D)**.

Since almost all UEA-1^+^ gill epithelial cells were M-type antigen-sampling cells, the fraction of MHC class II^+^ cells among UEA-1^+^ population was compared between mock-vaccination and *ex vivo*-vaccination. In the mock-vaccination, 17.7% of UEA-1^+^ cells showed MHC class II expression on their surface (Figure 5A), while the percentage of MHC class II^+^ UEA-1^+^ cells in the *ex vivo*-vaccination was 26.2% (Figure 5B). The difference was statistically significant between mock-vaccination and *ex vivo*-vaccination (*p* < 0.05, Figure 5C). The presence of UEA-1^+^ MHC class II^+^ cells in the gill epithelium after *ex vivo*-vaccination was confirmed by fluorescence microscopy (Figure 5D).

## Discussion

The mechanisms of antigen sampling in the mucosal epithelium of teleost fish are mostly unknown. In mammals, M cells in the gut mucosal epithelium take up antigens and deliver them to MALT, where antigen presentation by APCs occurs, and a local immune response is induced. Although teleost fish possess an acquired immune system, they lack highly organized lymphoid structures consisting of lymphoid follicles and germinal centers (8). In this study, we identified two phenotypes of gill epithelial antigen-sampling (GAS) cells in rainbow trout: M-type GAS cells with similar characteristics to mammalian M cells; and macrophage/DC-type GAS cells. Furthermore, we demonstrated that the M-type GAS cells show the potential for direct antigen presentation in the gill epithelium. These results suggest an unconventional system of antigen sampling and presentation in the teleost gill epithelium. Our findings may shed light on the mechanisms of local mucosal immune response induction in gill-breathing vertebrates.

It was shown in this work that M-type GAS cells are able to take up bacterins but not fluorescence microbeads during bath administration, suggesting a receptor-based mechanism. The engulfment of bacteria by M cells is dependent on the recognition of bacteria by several receptors. For example, mammalian M cells are able to take up wild-type *Escherichia coli* but not FimH-deficient *E. coli* since glycoprotein 2, a receptor expressed on M cells, selectively binds to FimH on the outer membrane of the bacteria (4). The gill epithelium is the major route for the entry of bacterins of species such as *Y. ruckeri* (21) and *V. anguillarum* (23) during bath vaccination. Immunohistochemistry analyses revealed that the gill epithelial cells actively sample vaccine antigens during bath vaccination (21, 23, 25, 27), while fluorescent microbeads administered by bath immersion are rarely taken up (28). These data suggest that M-type GAS cells take up antigens with a certain pattern through receptor-based mechanisms.

The M-type GAS cells showed similar characteristics to mammalian M cells, exemplified by their affinity for lectins (UEA-1^+^ WGA^−^) and by high expression of *Anxa5*. Other typical gene markers of mammalian M cells, such as *CCL20, Ctsh, Dnclc*, and *Gpx I* (29, 30), were also expressed in M-type GAS cells, although at similar levels to those in negative and macrophage/DC-type GAS cells, based on qRT-PCR analysis (data not shown). The α (1, 2) fucose carbohydrate moiety recognized by UEA-1 has been suggested to play a role in antigen trapping by M cells (31). A role for annexin V, which is important in endocytic transport and membrane scaffolding, has been suggested in M cell-mediated transcytosis (30). The gene expression patterns of M-type GAS cells suggest that they are closely related to mammalian M cells.

The main function of M cells is transcytosis of antigens to dendritic cells and macrophages in mammalian MALT (7). Although there are several reports suggesting that MHC class II is expressed on M cells (32, 33), the general view is that these cells are unable to process and present antigens to T cells since they are markedly deficient in lysosomes (34). In contrast, M-type GAS cells were found to exhibit small vacuoles in their cytoplasm and to express almost all genes related to the “phagosome,” “lysosome,” and “antigen processing and presentation” pathways. Further, MHC class II was constitutively expressed on a fraction of M-type GAS cells, which was significantly increased after bath vaccination, suggesting that the MHC class II expression is inducible by antigen stimulation. Similar to our results, MHC class II expression in the gill epithelium has also been reported in Atlantic salmon infected with *Neoparamoeba* sp. amoebae, although the corresponding cells have not been fully characterized (35). Thus, M-type GAS cells are more likely to present antigens in the gill epithelium than bona-fide mammalian M cells in the intestinal epithelium.

In mammals, cells other than M cells in mucosal tissues are able to take up antigens. These cells include type II alveolar epithelial cells, keratinocytes and goblet cells. Multiple reports suggest that most of these epithelial cells show constitutive MHC class II expression and present antigens to T cells (36). Debbabi et al. (37) reported that type II alveolar epithelial cells present exogenous antigens from *Mycobacterium tuberculosis* through MHC class II and that they can prime antigen-specific CD4^+^ T cells. Keratinocytes are also able to efficiently process and present soluble antigens only to CD4^+^ memory T cells, resulting in Th1 and Th2 cytokine secretion (38). However, in the present study, no genes characteristic of type II alveolar epithelial cells and keratinocytes, such as surfactant protein A, cytokeratin-8, keratin-6, and aquaporin-3 (39), were found in the mRNA library from M-type GAS cells. Due to the unique gene expression pattern observed in this phenotype of GAS cells and the fact that this cell population constitutively expresses MHC class II in teleosts, M-type GAS cells should be a different cell type from mammalian intestinal M cells.

It has been shown in the past that after bath vaccination with bacterins, the corresponding antigens are transported to the head kidney, trunk kidney and spleen via the blood circulation (21). It has been proposed that vaccine components are processed primarily at the site of melano-macrophage centers in lymphoid organs (40). The spleen and head kidney are the major lymphoid organs of teleost fish (41), and their melano-macrophage centers are considered to be the phylogenetic precursors of germinal centers in higher vertebrates (42). Mammalian macrophages and dendritic cells play an important role in antigen transportation. Rességuier et al. (43) showed that dendritic cells take up surfactant-free poly (lactic acid) nanoparticles in the gill epithelium. In the present work, numerous gill epithelium-resident macrophage/DC-like GAS cells that contained bacterin were found following bath vaccination. This second type of GAS cells might be important for the transportation of vaccine antigens into the lymphoid organs and for the subsequent induction of systemic immune responses, such as the production of specific antibodies (44).

Antigen-specific mucosal immune responses have been frequently reported in mammals (45). Recently, Xu et al. (46) further showed that rainbow trout survived an infection with the parasite *Ichthyophthirius multifiliis* by inducing local proliferation of IgT^+^ B cells and IgT production in the gills. In addition, Kai et al. demonstrated that high IgT levels are induced by bath and immersion vaccination, whereas injection vaccination induces high IgM levels (47). These reports suggest that distinct local mucosal immune responses have evolved during the course of vertebrate evolution. Here, we suggest that teleost M-type GAS cells represent a primitive type of epithelial antigen-sampling cells. Bona-fide M cells are critical in terms of inducing local mucosal immune responses in mammalian MALTs, mainly via the transcytosis of antigens (7). Teleost M-type GAS cells may play a similar role in the phylogenetically primitive teleost immune system. In addition, the presence of MHC class II expression suggests an additional role in antigen presentation in the gills, which are an organ with high T cell abundance, especially in ILT (9, 10). Moreover, the results of this work may be valuable for the development of mucosal vaccines that specifically target GAS cells. Due to the rapidly growing aquaculture industry and declining capture fishery resources, effective mucosal vaccines for farmed fish are urgently needed. Bath vaccines significantly reduce working costs and the stress that is usually induced by vaccination via injection of individual fish.

## Availability of data and material

The whole transcriptome sequence raw data were deposited in the DDBJ Sequence Read Archive (Accession Number: DRA006692).

## Author contributions

GK and UF designed the study. GK wrote the manuscript. GK, UF, HM, YN, YI, and TY performed the experiments. HK analyzed the RNA-seq data. MS, UF, and TY edited the manuscript.

### Conflict of interest statement

The authors declare that the research was conducted in the absence of any commercial or financial relationships that could be construed as a potential conflict of interest.
