# Supplementary material for: A Novel Antigen-Sampling Cell in the Teleost Gill Epithelium With the Potential for Direct Antigen Presentation in Mucosal Tissue
Source: Front Immunol. 2018 Sep 20;9:2116. doi: 10.3389/fimmu.2018.02116 (PMC6158387; doi:10.3389/fimmu.2018.02116)
Supplement: Supplementary file 1 [file Data_Sheet_1.DOCX]

**Supplementary Information**

**A novel antigen-sampling cell in the teleost gill epithelium with the potential for direct antigen presentation in mucosal tissue**

Goshi Kato^1^*, Haruya Miyazawa^1^, Yumiko Nakayama^1^, Yuki Ikari^1^, Hidehiro Kondo^1^, Takuya Yamaguchi^2^, Motohiko Sano^1^ & Uwe Fischer^2^

^1^Department of Marine Biosciences, Tokyo University of Marine Science and Technology, Tokyo, Japan

^2^Institute of Infectology, Friedrich-Loeffler-Institut, Federal Research Institute for Animal Health, Greifswald-Insel Riems, Germany

*Correspondence should be addressed to G.K. ([gkato00@kaiyodai.ac.jp](mailto:gkato00@kaiyodai.ac.jp))

Address: Konan 4-5-7, Minato, Tokyo 108-8477, Japan

TEL: +81-3-5463-0462

E-mail: gkato00@kaiyodai.ac.jp

This file includes:

**Supplementary Figures and Tables (Fig S1 to S4 and Table S1 to S3)**

**Supplementary Materials and Methods**

**Supplementary References**

**Fig S1** The two phenotypes of antigen-sampling cells did not react with mAbs against rainbow trout CD8α, IgM, or thrombocytes. (A) Flow cytometry of gill mucosal cells that had taken up *Ass* bacterin after bath vaccination. (B-D) Cells that had taken up *Ass* bacterin were gated, and their staining pattern for CD8α is shown in *B*; for IgM in C; and for thrombocytes in D. White areas show the cells that were not treated with mAbs (conjugate control), and gray areas show mAb-positive cells. Experiments were done by in vivo bath vaccination. Data are representative of three experiments.

A B


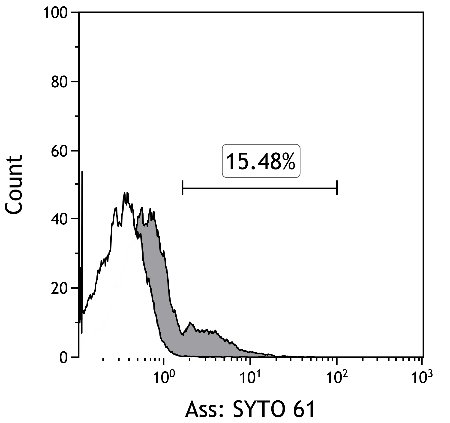

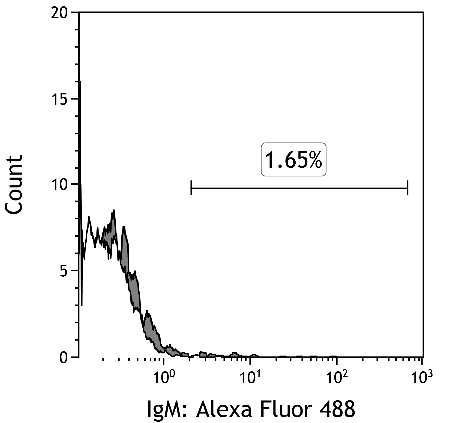


C D


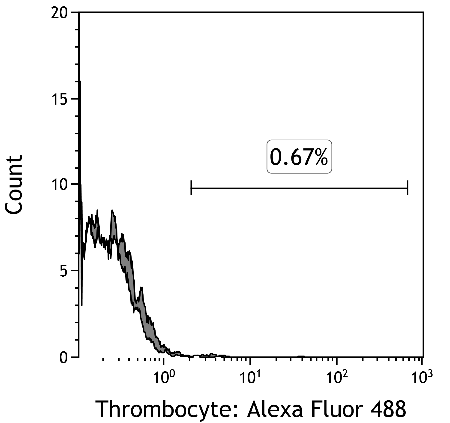

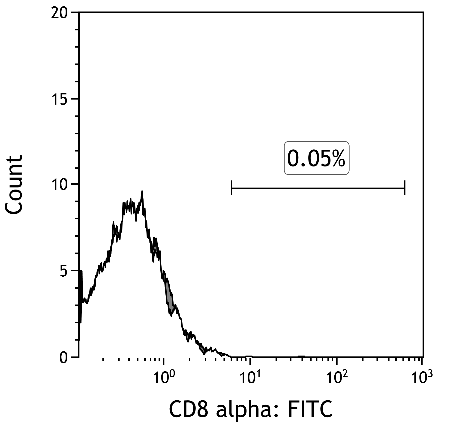


**Fig S2** Flow sorting of UEA-1^+^ *Ass*^+^ cells, UEA-1^-^ *Ass*^+^ cells, and negative cells from the gill outer layer cells of fish bath vaccinated with *Ass* bacterin. (A-D) Dot plots depict UEA-1 against *Ass* bacterin staining of gill mucosal cells in A, sorted UEA-1^+^ *Ass*^+^ cells in B, sorted UEA-1^-^ *Ass*^+^ cells in C, and sorted negative cells in D. Experiments were done by in vivo bath vaccination. Data are representative of three experiments.

A B


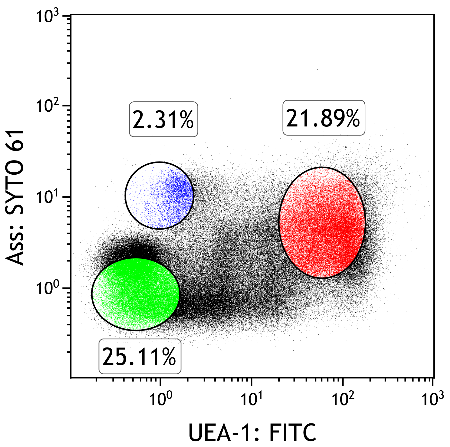

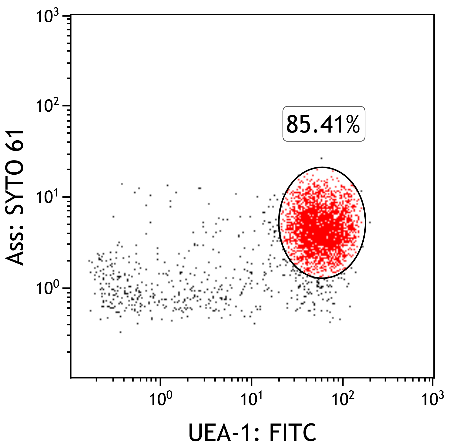
 **
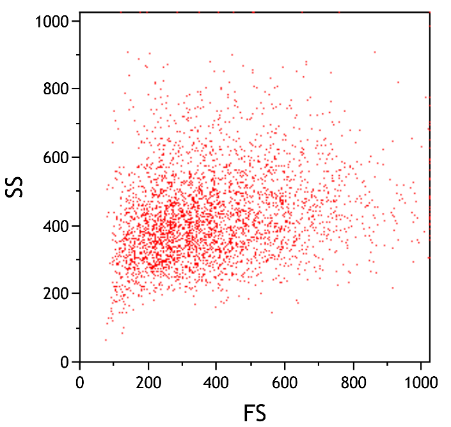
**

C


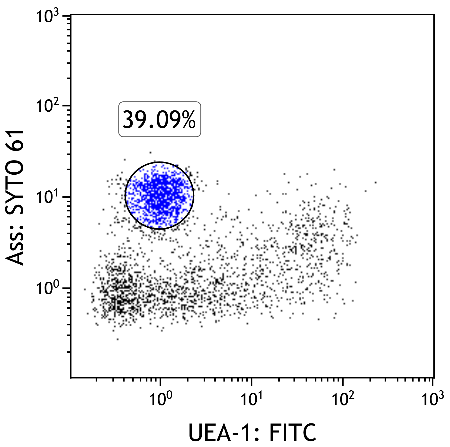
 **
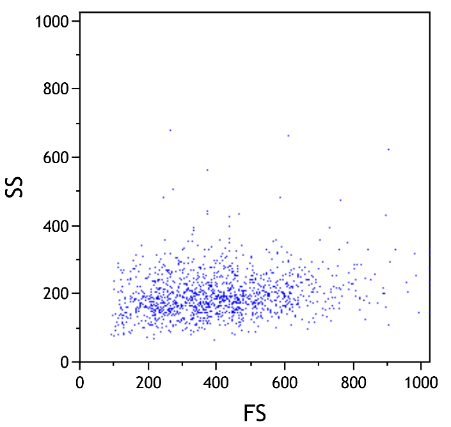
**

D


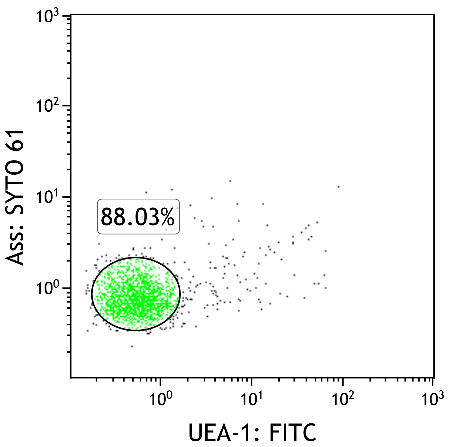
 **
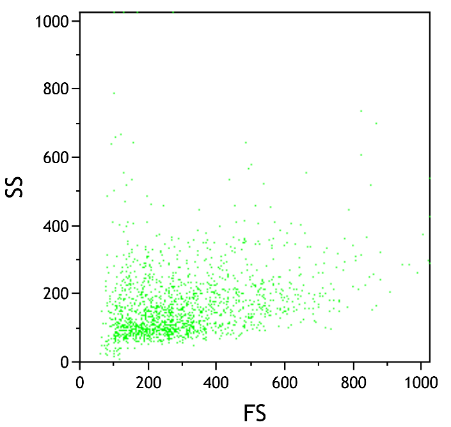
**

**Fig S3** KEGG pathway maps for orthologues found in the mRNA library of the UEA-1^+^ *Ass*^+^ cell population. (A) Lysosome pathway (ID = 04142), (B) phagosome pathway (ID = 04145), and (C) antigen processing and presentation pathway (ID = 04612). Boxes highlighted in green indicate orthologues in rainbow trout involved in the respective pathways. Data are from one experiment.

A B


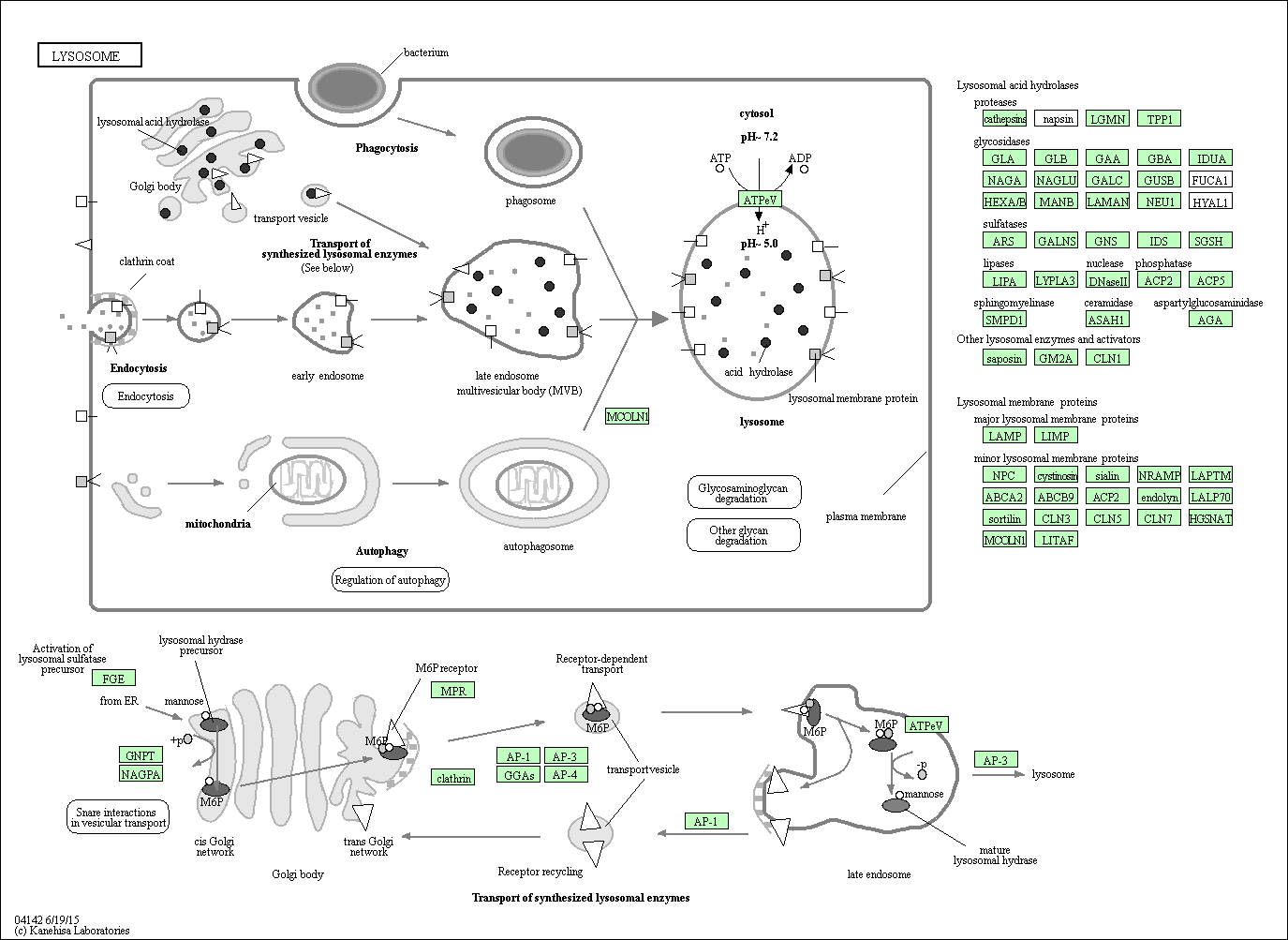

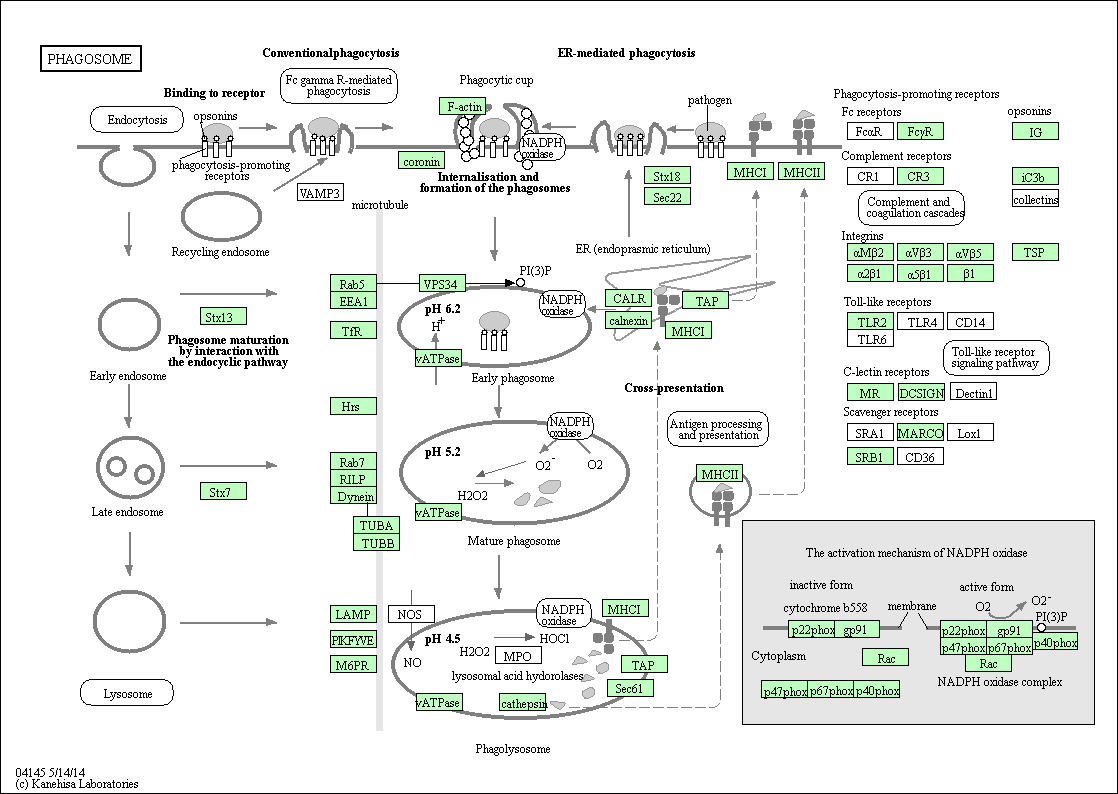


C


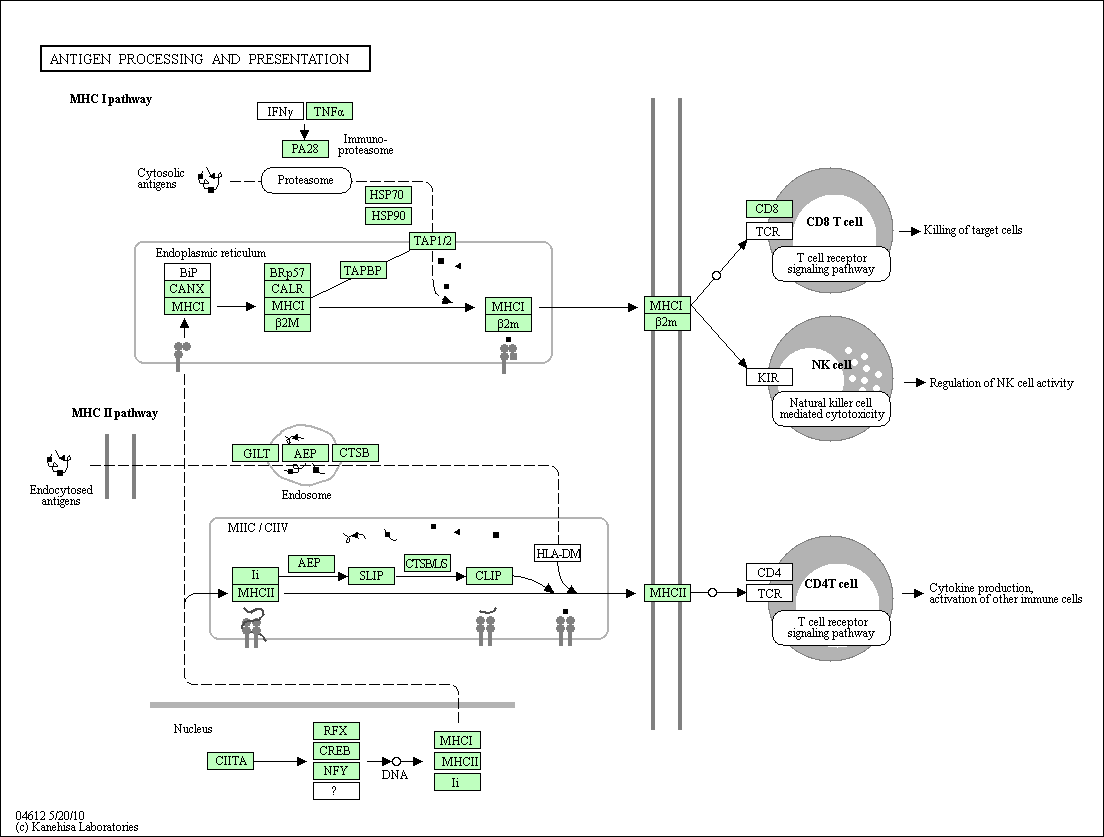


**Fig S4** KEGG pathway maps for orthologues found in the mRNA library of the UEA-1^-^ *Ass*^+^ cell population. (A) Lysosome pathway (ID = 04142), (B) phagosome pathway (ID = 04145), and (C) antigen processing and presentation pathway (ID = 04612). Boxes highlighted in green indicate orthologues in rainbow trout involved in the respective pathways. Data are from one experiment.

A B


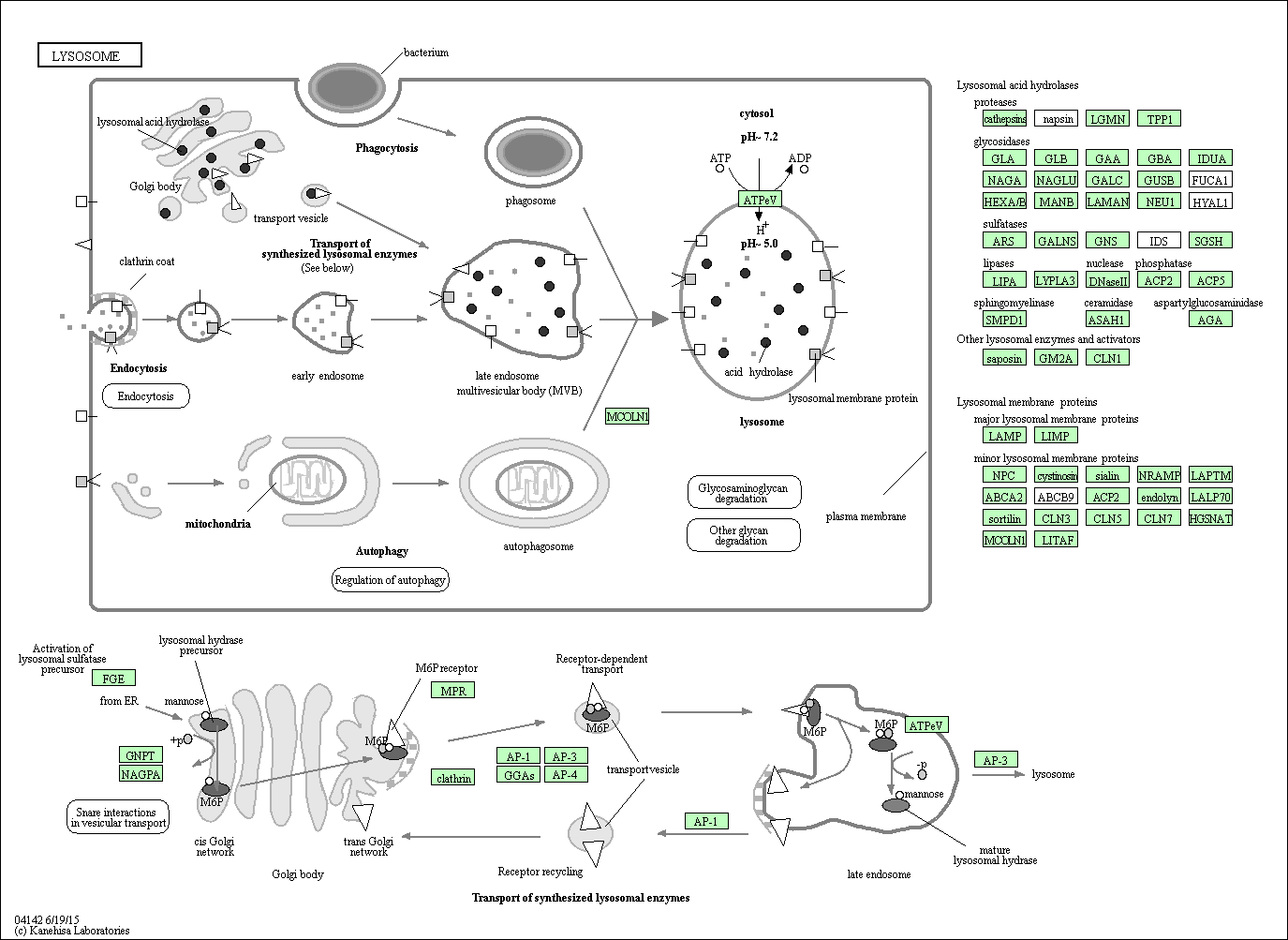

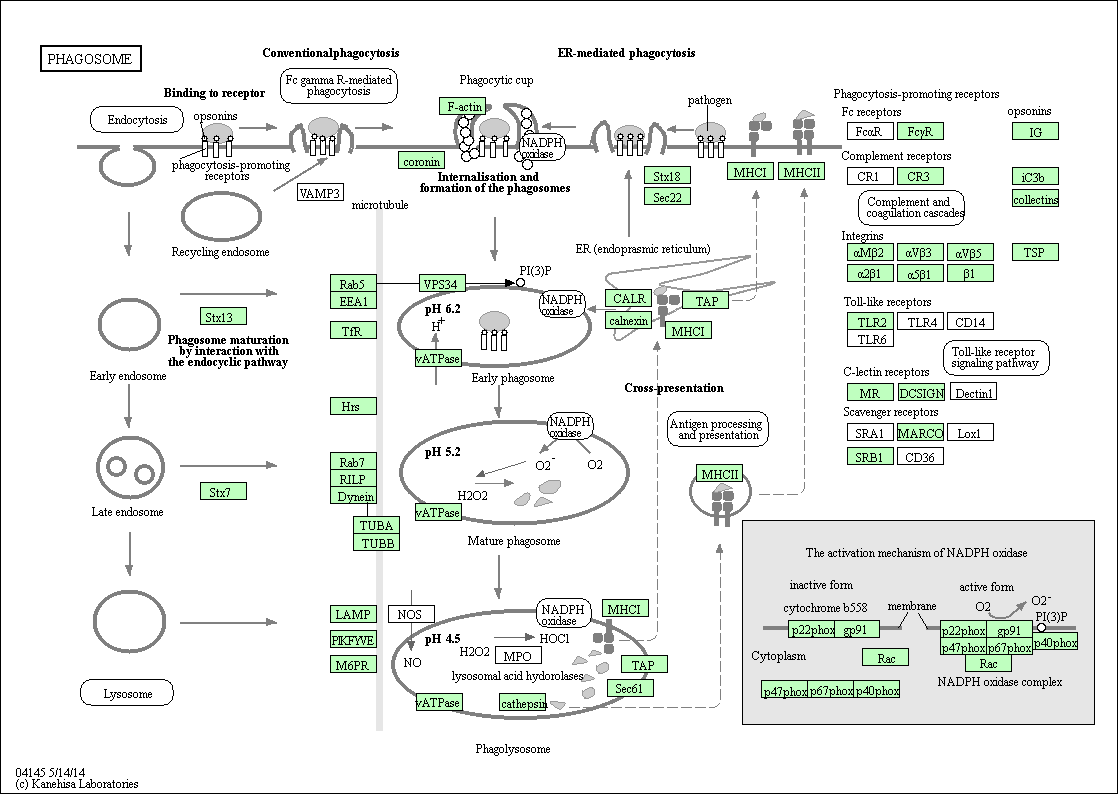


C


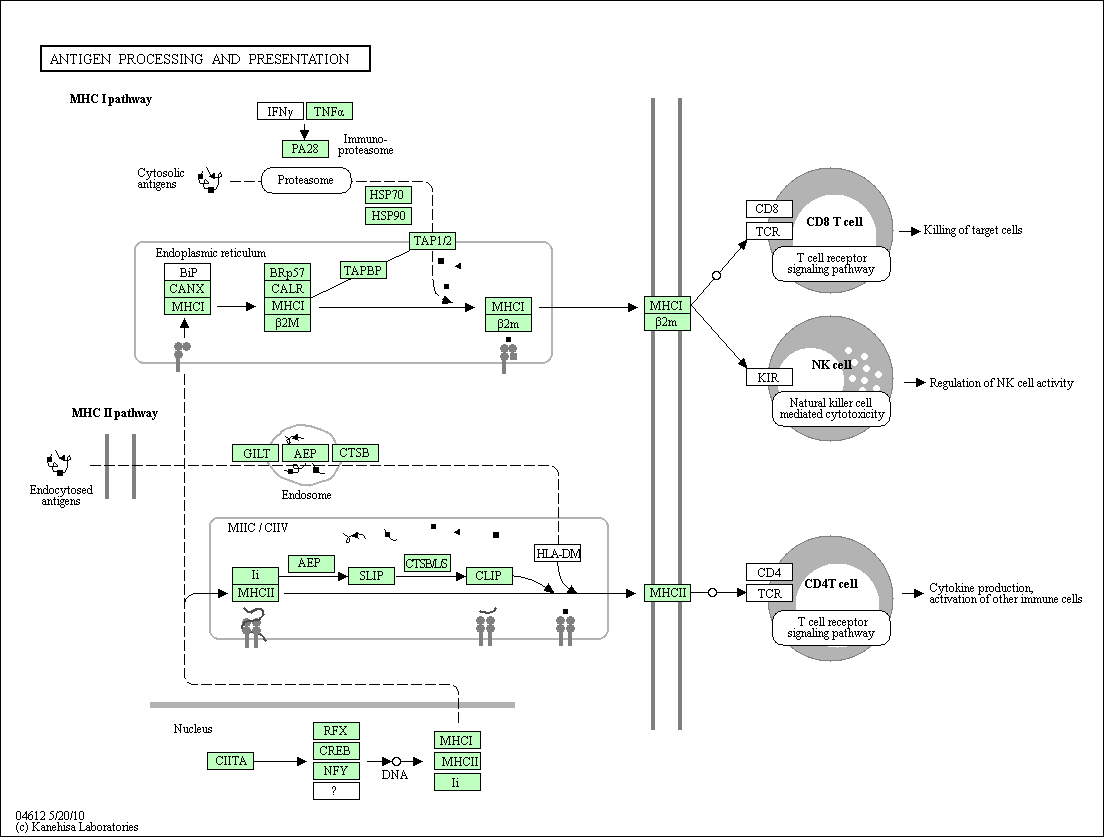


**Table S1** Statistics for Illumina Miseq sequencing liblaries

|  | Nucleotide length |
| --- | --- |
| Sequencing  Estimated Yield (Mbps)  Reads  Total Reads DP (GAS)  Total Reads SP  Total reads Negative  Contig  Total Trinity transcripts  Median contig length  Average contig length  Total assembled bases  Contig N50 | 4392.1  5,838,938  5,819,270  783,134  174,245  349  622  108,456,943  943 |

**Table S2** KEGG Pathway related to immune system, transport and catabolism in each library

| KO  number | Pathway | The number of unigenes | | |
| --- | --- | --- | --- | --- |
|  |  | Negative | UEA-1^+^ Ass^+^ | UEA-1^-^ Ass^+^ |
| 04010  04060  04062  04064  04142  04144  04145  04151  04612  04620  04621  04622  04623  04810 | MAPK signaling pathway  Cytokine-cytokine receptor interaction  Chemokine signaling pathway  NF-kappa B signaling pathway  Lysosome  Endocytosis  Phagosome  PI3K-Akt signaling pathway  Antigen processing and presentation  Toll-like receptor signaling pathway  NOD-like receptor signaling pathway  RIG-I-like receptor signaling pathway  Cytosolic DNA-sensing pathway  Regulation of actin cytoskeleton | 76  44  50  40  45  97  44  82  23  31  19  22  18  62 | 119  88  83  63  92  152  68  149  31  57  35  43  39  99 | 132  92  86  68  91  155  73  152  30  60  35  42  38  105 |

**Table S3** Primers used for real-time PCR

| Primer name | Nucleotide sequence 5’-3’ |
| --- | --- |
| *Anxa5*-F  *Anxa5*-R  *Cldn3*-F  *Cldn3*-R  *CDH1-*F  *CDH1-*R  *KRT13-*F  *KRT13-*R  *KRTE1-*F  *KRTE1-*R  *IL17R*-F  *IL17R*-R  *CD83*-F  *CD83*-R  *IL-1β 3*-F  *IL-1β 3*-R  *IL-12 p40b*-F  *IL-12 p40b*-R  *PTPRC-*F  *PTPRC-*R  *MHC-IIB*-F  *MHC-IIB*-R  *MHC-II Ii*-F  *MHC-II Ii*-R  *EF1α*-F  *EF1α*-R | CCTGAAGGAGCGATACCTGGATAGA  TTGGGTTACACAGGACTCAGAGAGT  GGATGAGGCTTCCAAAGCTAAAGTA  AGAGGGTTGTAAAAATCCCTGATGA  CATGGTTGTCCTGAGAGTGTCTGAC  AATTCCCAGAATGCTAGAGATGCCG  AACGGAGGCATCTATCTCAGCATTG  CAATGTCTGCCTCTACTGACTGACG  CGAGTCCTGGTACAAGCAGAAGTTT  TGACGTTCTCGATCTCGTTTTGGAG  CATACGTTGTCTCAGTCTCCAACCT  ATCTTTACAATCGGGCACAGTGATA  GCCCTCTAATAAGGAGTCTGGCCTA  CATCAGCCAAAAGTTCCACTTCACG  GCCAGTTGTCTTAGGGATTGACCAA  CTTACAGCGCTCCAACTGTAGGAC  GGTGGATCGAAAGGTCTTCACTGAG  TGTTTTCTAGACGCTCCTTGCCATT  ATGACAAAGCTGTGATTGGGTTCCT  ATCATTGTTGTTGGACTTCTTGCGC  ATCTCAGATTCAACAGCACTGTGGG  CGTTAGGCTTACATAGACGCTCCAG  AAGATAGCAGGGTTCACAGTGTTGG  GCTTGCGCATGTTATCATTGGTCTT  ATCACTGGTACCTCTCAGGCTGATT  CAATGAGCTGTTTCACTCCCAGAGT |

**Supplementary Materials and Methods**

**Flowcytometry with mAbs against rainbow trout CD8α, IgM, or thrombocytes**

After in vivo bath vaccination with SYTO 61-stained Ass, gills were washed twice with cell culture medium. For separation of epithelial cells, the gills were incubated in PBS containing 10 mM EDTA with a stirring for 20 min at 4°C. The cells were then washed three times, suspended in cell culture medium and stained with a CD8α (1), IgM (2), or thrombocytes (3) as previously described. Goat anti-mouse IgG (H+L) Alexa Fluor 488 (1 µg/ml, Life Technologies) and goat anti-rat IgG (H+L) FITC (4 µg/ml, Life Technologies) were used as secondary antibodies. After being washed three times, the cells were re-suspended in cell culture medium and stained with propidium iodide (2 μg/ml, Life Technologies). Flow cytometry analysis was performed using a FACSCalibur flow cytometer (BD Biosciences). Data from the cytometers were analyzed with Kaluza Flow Cytometry Analysis Software (Beckman Coulter).

**Supplementary References**

1. Takizawa F, et al. (2011) The expression of CD8α discriminates distinct T cell subsets in teleost fish. *Dev. Comp. Immunol.* 35: 752–763.
2. Deluca D, Wilson M, Warr GW (1983) Lymphocyte heterogeneity in the trout, Salmo gairdneri, defined with monoclonal antibodies to IgM. *Eur. J. Immunol.* 13: 546–551.
3. Köllner B, Fischer U, Rombout JH, Taverne-Thiele JJ, Hansen JD (2004) Potential involvement of rainbow trout thrombocytes in immune functions: a study using a panel of monoclonal antibodies and RT-PCR. *Dev. Comp. Immunol.* 28: 1049-1062.
